# ‘Visual’ Acuity of the Congenitally Blind Using Visual-to-Auditory Sensory Substitution

**DOI:** 10.1371/journal.pone.0033136

**Published:** 2012-03-16

**Authors:** Ella Striem-Amit, Miriam Guendelman, Amir Amedi

**Affiliations:** 1 Department of Medical Neurobiology, Faculty of Medicine, The Institute for Medical Research Israel-Canada, The Hebrew University of Jerusalem, Jerusalem, Israel; 2 The Edmond and Lily Safra Center for Brain Sciences (ELSC), The Hebrew University of Jerusalem, Jerusalem, Israel; University of Bologna, Italy

## Abstract

Sensory Substitution Devices (SSDs) convey visual information through sounds or touch, thus theoretically enabling a form of visual rehabilitation in the blind. However, for clinical use, these devices must provide fine-detailed visual information which was not yet shown for this or other means of visual restoration. To test the possible functional acuity conveyed by such devices, we used the Snellen acuity test conveyed through a high-resolution visual-to-auditory SSD (The vOICe). We show that congenitally fully blind adults can exceed the World Health Organization (WHO) blindness acuity threshold using SSDs, reaching the highest acuity reported yet with any visual rehabilitation approach. This demonstrates the potential capacity of SSDs as inexpensive, non-invasive visual rehabilitation aids, alone or when supplementing visual prostheses.

## Introduction

Blindness is a highly limiting disability, affecting tens of millions of individuals worldwide [1]. One of the current challenges in sight restoration and sensory aids for the blind pertains to the possible visual acuity and capacity which can be transmitted through various restoration approaches. For example, neuroprostheses [2], [3] which offer great hope for restoring visual qualia suffer at the moment disadvantages such as invasiveness, their restricted applicability to particular etiologies, extremely high cost and poor resolution and visual field to date (e.g. maximal resolution of 60 electrodes and 20° visual field-of-view in chronic implantation clinical trials; Second Sight Inc., Sylmar, CA, USA; http://2-sight.eu/ee/benefits-of-argus-ii; and 1000–1500 electrodes and 11° visual field-of-view in development stages [4]; Retina Implant AG, Reutlingen, Germany). Moreover, the resulting acuity is lower than predicted given the number of pixels, because the translation from technical resolution to functional acuity is highly complex. For instance, the newest subretinal prosthesis under development technically has 1500 pixels, but provides a much lower than expected functional acuity, with a maximal measurable acuity of only 20/1000 [4]; thus the smallest letter implant patients can see at 20 feet could be seen by a normal eye at 1000 feet (i.e. they can discern only extremely large letters).

Visual rehabilitation may alternatively be achieved using Sensory Substitution Devices (SSDs [5]) which enable the blind to ‘see’ using their other senses. Initially these focused on tactile-to-visual SSDs [6], and interestingly, although their maximal technical resolution was only 144 pixels at the time, they enabled better acuity than the highest 1500-electrode technical resolution retinal implant under development today (20/860 vs. 20/1000 [4], [7], [8]), stressing the need to test for functional acuity beyond potential pixel resolution. However, this acuity was still functionally quite poor. For purposes of comparison, the blindness threshold of the World Health Organization (WHO) is set at best corrected sight of 20/400 acuity (and a 10° visual field; 10^th^ revision of the WHO international classification of diseases, update 2007; note that other legal definitions may be applicable in various countries), and up to now retinal prostheses and tactile-to-visual SSDs remain far below such levels of acuity.

**Figure 1:**
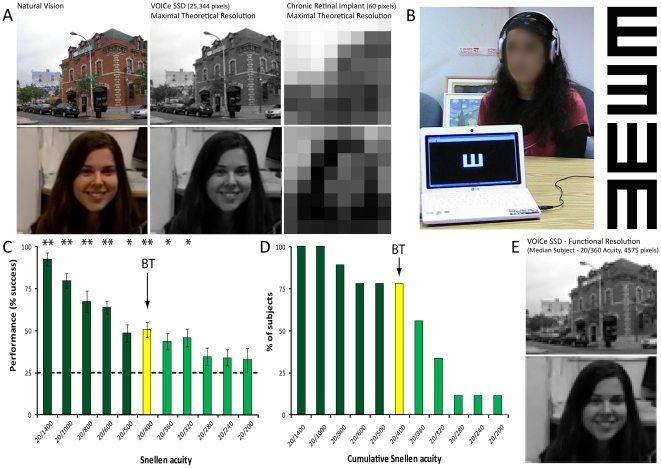
Visual Acuity of the congenitally blind using a visual-to-auditory SSD. A. Illustration of the typical theoretical resolution (in terms of number of pixels) provided by different current means of visual rehabilitation (the vOICe and implants provide only gray scale information). B. A blind participant during training, perceiving an image of a large Snellen E and identifying its direction (of the 4 possibilities). C. The group performance on the Snellen acuity test. * p<0.01, ** p<0.001. As an interesting reference point in relation to visual acuity in health and disease, we also display the World Health Organization (WHO) criterion for blindness, at an acuity of 20/400, in the results (yellow bar, BT – WHO Blindness Threshold). D. Cumulative frequency Snellen acuity of the individual participants; the percentage of subjects whose visual acuity threshold passed each acuity level. Most (5/9) participants performed above chance level even above the 20/400 WHO BT. E. The images in A are processed to reflect the functional resolution achieved in this experiment by the median participant (Snellen acuity of 20/360, below blindness threshold). This resolution enables identification of the scene and, at least in one of our subjects, emotional facial expression in a real life scenario (see **Movie S1, Figure S1**).

In contrast, auditory SSDs can offer, at least theoretically, extremely high resolution. While one such device used in research and in an effort to rehabilitate the blind, the PSVA (Prosthesis Substituting Vision with Audition; [9]) has a maximal theoretical resolution of only 124 pixels (although this too has been shown to enable some functional sight; [10]), “The vOICe” SSD [11] can in principle generate much higher resolution, up to 25,344 pixels (see **Figure 1A**). However, its actual functional visual acuity has never been tested to the best of our knowledge, and especially not in a blind users group systematically. It is thus important to determine the best possible visual acuity that can be achieved by blind individuals using such an auditory SSD, in order to understand the potential value of these devices. Moreover, as critical developmental periods [12], [13], [14] for perception of natural vision in adulthood may limit the medical means of sight restoration (for example, several rare accounts of sight restoration in adulthood resulted in only partially functional vision, likely due to such limitations; [15], [16], [17], [18], [19], [20]), it is interesting to determine if early-onset and congenitally blind adults can learn to see fine “visual” details after many years of blindness using SSDs.

To clarify these questions we devised an adapted visual-to-auditory version of the Snellen E-chart visual acuity test used by ophthalmologists (See **Figure 1B**), and used it to test a group of eight congenitally and one early-onset fully blind individuals (see **Table 1**) who were given structured relatively short (tens of hours; see details in the experimental procedures) training in vOICe visual perception.

**Table 1:** Characteristics of blind participants.

Subject	Age & Gender	Cause of blindness	Light perception	Handedness	Blindness onset	Training duration (hours)	
1	27 F	Retinopathy of prematurity	None	Right	birth	55	
2	23 F	Microphthalmia	None	Right	birth	65	
3	23 F	Leber congenital amaurosis	Faint	Ambidextrous	birth	60	
4	24 F	Retinopathy of prematurity	None	Right	birth	61	
5	30 M	Retinopathy of prematurity	None	Right	birth	101	
6	33 F	Enophthalmia	None	Left	birth	32.5	
7	48 M	Retinopathy of prematurity	None	Right	birth	101	
8	21 F	Retinopathy of prematurity	None	Right	birth	87	
9	22 F	Microphthalmia, Retinal detachment	None	Left	birth	98	

## Methods

### Participants

Eight congenitally and one early-onset fully-blind individuals participated in the experiment (see **Table 1**). All participants had normal hearing, and had no neurological or psychiatric conditions. The Hebrew University's ethics committee for research involving human subjects approved the experimental procedure and written informed consent was obtained from each participant.

### Visual-to-auditory sensory substitution

The vOICe [11] is a visual-to-auditory sensory substitution device (SSD) which converts images into sounds, technically preserving visual detail at high resolution (up to 25,344 pixels, the resolution used here; see **Figure 1A**). In a clinical or everyday setting, users wear a video camera connected to a computer and stereo headphones; the images are converted into “soundscapes” using a predictable algorithm, allowing them to listen to and then interpret the visual information coming from a digital video camera. Remarkably, proficient users are able to differentiate the shapes of different objects, identify the actual objects, and also locate them in space [21], [22], [23]. The functional basis of this visuo-auditory transformation lies in spectrographic sound synthesis from any input image, which is then further perceptually enhanced through stereo panning and other techniques. Time and stereo panning constitute the horizontal axis in the sound representation of an image, tone frequency makes up the vertical axis, and loudness corresponds to pixel brightness.

### Training procedure

All the participants in this study were enrolled in a new unique training program in which they were taught how to effectively extract and interpret high-resolution visual information from the complex soundscapes generated by the vOICe SSD. Each participant was trained for several months in a 2-hour weekly training session by a single trainer on a one-by-one basis. The training duration and progress rate varied across participants and were determined by the personal achievements and difficulties (the average training here was 73 hours, for detail of individual training durations see **Table 1**). The training program was composed of two main components: structured 2-dimensional training, in which the participants were taught how to process 2-dimensional still (static) images, and live-view training in which visual depth-perception and training in head-‘eye’ (camera installed on glasses)-hand coordination were taught, using a mobile kit of the vOICe SSD assembled in our lab. In the structured 2-dimensional training the participants were taught guiding principles of visual processing by learning to process hundreds of images of seven structured categories: geometrical shapes, Hebrew letters and digital numbers, body postures, everyday objects, textures (geometrical shapes placed over visual texture, used to teach object-background segregation), faces and houses, introduced in controlled and growing complexity and detail.

### Experimental design

We conducted a Snellen tumbling-E test, which is used by ophthalmologists to measure visual acuity. The Snellen fractions are measures of the spatial acuity of sight (if vision is blurred in a given size, the orientations cannot be reported). The original ophthalmologists' Snellen tumbling-E test used to measure visual acuity contains rows of the letter E in various types of rotation (up, down, left or right), and the patient is asked to state where the limbs of the letter “E” are pointing. Depending on the smallest letter line (i.e. the smallest size) the patient can read or recognize the orientation, his visual acuity is defined. The Snellen fractions are measures of visual spatial discrimination, relating to the ability to identify small high-contrast letters at a specific distance. In “20/20 vision” (20 feet or 6/6 in the metric system) the numerator refers to the distance in feet (or meters) between the subject and the chart, the denominator is the distance at which the lines that make up the letters are separated by a visual angle of 1 arc minute (minute of angle), which is the level of discrimination achieved by an eye with no refractive errors, or with the errors corrected. To control for individual factors which could affect the performance of our participants other than pure perceptual acuity, we introduced the tumbling E stimuli of the Snellen acuity chart using static images of each differently oriented E separately (see in **Figure 1B**), similarly to previous testing of a visual-to-tactile SSD ([7]; with which an acuity of 20/860 was achieved). Stimuli were created by photographing a standard Snellen chart with a 66° field-of-view webcam (A4Tech, Montclair, CA, USA) from a distance of 1 meter, and calculating the Snellen fraction from this distance according to the standard reference scale. In this way we created a series of stimuli in different orientations and with different acuity scores (see **Table 2**), within a relatively wide field-of-view (thus not trading high acuity for a small field-of-view). Prior to testing, the subjects were trained for one hour on the Snellen acuity task, in order to familiarize them with the task and response buttons. Subjects were introduced to tumbling E's in all four directions at large sizes (larger than tested in the experiment) and were trained to identify the letter directions. The order of the stimuli in the training did not reflect that in the test, which was differently randomized per stimulus size.

**Table 2:** Snellen stimuli sizes.

Snellen acuity (m)	letter size (mm)	LogMAR
20/2000	146	2
20/1800	131	1.954
20/1600	117	1.903
20/1400	102	1.845
20/1200	88.7	1.778
20/1000	73	1.699
20/800	58	1.602
20/600	44	1.477
20/500	36	1.398
20/400	29	1.301
20/360	26	1.255
20/320	23.5	1.204
20/280	20.4	1.146
20/240	17	1.079
20/200	15	1

Snellen original stimuli sizes are reported in Snellen fractions (distance from which the participant perceives the letter in meters in the numerator and the distance from which a normally sighted individual would perceive the same letter in the denominator), physical letter size (in mm) and logMar, a linear scale which expresses the logarithm of the minimal angle of resolution [24].

During the experiment, soundscape stimuli were played using the Presentation software (Neurobehavioral Systems, CA, USA) in a pseudo-randomized order of E directions for each size, in decreasing order of size, similar to a conventional eye exam. Each size was presented four times in each of the four directions. Each soundscape was played until the subject responded regarding its position using a keyboard by pressing the arrow in the corresponding direction. No “zoom in” of the soundscapes was permitted, thus the field-of-view was fixed during the entire experiment to represent a 66° visual field (much more than the WHO blindness threshold for field of view, which is 10°). The answer and the reaction time were recorded, and no feedback was given to the subject during the experiment. Average reaction time per stimulus (across the stimuli sizes) was 9.16±1.26 seconds, and no significant correlation was found between reaction time and acuity (in linear LogMAR units, see **Table 2**; [24]) or success rate (participants were not instructed to answer as quickly as possible, thus a speed-accuracy tradeoff was not necessarily expected).

## Results

We analyzed the data both statistically at the group level (providing the raw accuracy scores of the group, **Figure 1C**) and at the single-subject level of individual acuity scores (**Figure 1D**).

Group performance differed statistically from chance level at all visual acuities up to 20/320 (one-way ANOVA; p<0.01; See **Figure 1C**), below the WHO blindness criterion (20/400). In addition, individual ‘visual’ acuity scores were determined by the smallest size at which a participant achieved over 60% correct responses, more than twice the chance level on this task (25% correct responses). The visual acuity of the individual participants varied between 20/200 and 20/600 (see **Figure 1D** for a cumulative acuity distribution). Therefore, all the participants performed better than reported using tactile SSD (20/860; [7], [8]) and the highest-resolution retinal prostheses (20/1000; [4]). Interestingly, five of the nine participants (55%) had visual acuity that exceeded the visual acuity threshold for blindness as defined by the WHO.

## Discussion

Our findings suggest that early and congenitally blind individuals using auditory SSDs can retrieve detailed visual information at a much higher resolution than previously demonstrated with any other sight rehabilitation approach. Most of our participants could even pass the WHO blindness threshold (also demonstrated statistically for the whole group) when using a SSD with a relatively wide field-of-view, capturing more than half of the binocular horizontal visual field in humans [25] (66°; it is important to note that no use of “zoom-in” was allowed in the experiment, which could enhance performance even further but at the expense of the field-of-view) and at least formally by the WHO criteria, be defined as low-vision sighted. For a demonstration of the possible detail conveyed at the resolution perceived by our median participant, see **Figure 1E** which roughly corresponds to extracting information from ∼4500 of the ∼25,000 pixels transmitted by The vOICe. This is by no means the upper bound as it may well be that further specific high-acuity training will yield better acuity results. These results show that high visual acuity can be restored to early-onset and congenitally blind individuals even after decades of (or life-long) blindness, suggesting there may be adult plasticity at the most important level – of actual “visual” perception in the adult congenitally blind. Retrieving high-acuity information from sounds may be more difficult and slower than real vision in more complex, natural settings. However, some capabilities demonstrated by our participants during training suggest this too may be possible. For example, our participants were able to identify and mimic the body posture of a person standing a few meters away, navigate in crowded corridors while avoiding obstacle, and recently, one of the participants in our study (participant #4, who achieved a 20/400 acuity score) was also able to identify live, 3-dimentional emotional facial expressions (see **Movie S1, Figure S1**).

Although our study did not inspect the SSD visual acuity of normally sighted or that of late-onset blind individuals, one may expect that they will show comparable performance, though possibly with some inferiority relative to early-onset blind, due to the latter's compensatory advantage in auditory processing [26], [27], [28]. While future studies should test this hypothesis more rigorously, we also trained in vOICe-use a few sighted individuals (though to a more limited extent), who had no difficulty in learning to apply the SSD transformation algorithm or in extracting high-detail information from soundscapes, supporting the usability of this system also for late-onset blind.

Therefore, SSDs may be beneficial in restoring high-resolution functional vision at very low cost (the vOICe software is free to blind users; the setup costs approximately $200 or much less if utilized through existing android cellphones). The factor of price may prove important to the vast majority of the world's visually impaired population, amounting to tens of millions of individuals worldwide, who reside in developing countries (about 90% of the world's visually impaired live in developing countries; [1]) and are unlikely to benefit in the near future from expensive medical equipment. In fact, even in developed countries not all types of blindness will be treatable in the coming years using prostheses, as these implants currently depend upon the existence of intact retinal ganglion cells, which characterize only some (such as age-related macular degeneration and retinitis pigmentosa) but not all blindness etiologies [3]. Moreover, SSDs may also be beneficially used as a complement to visual prostheses or other novel medical advances [2], [4], [29], [30] in developed countries (and later in the rest of the world). SSDs may be used for instance before a retinal prosthesis implantation, to train the visual cortex to ‘see’ again after years or life-long blindness, by addressing and strengthening the preserved “visual” task selectivities of the occipital cortex of the blind; for instance we recently showed that SSD use activates the ventral and dorsal streams respectively [31] (see also [21], [32], [33], [34], [35], [36], [37], [38]), and to teach visual processing principles (such as visual monocular depth cues [10]) that were not in use for extended periods prior to the operation. This training might be important not only for understanding high-acuity and holistic vision again based on a smaller number of pixels (as provided by retinal prostheses, at least currently), but also to awaken the ‘visual’ system to performing its original tasks [39], [40]. SSDs can also be used post- surgically, to provide parallel explanatory “sensory interpreter” input to the visual signal arriving from the foreign invasive device (early-onset blind may otherwise find it difficult to interpret vision; [15], [16]). At a later stage the SSD can be used to provide information beyond the maximal capabilities of the prostheses, increasing both the resolution (as shown here) and the visual field-of-view (which is also currently very limited in retinal prostheses). SSDs can additionally be used for visual perception enhancement for individuals who have impaired natural vision, either in terms of acuity (for example in cases of cataract) or reduced visual field (such as that affecting retinitis pigmentosa or glaucoma patients).

In discussing SSDs benefits, visual-to-auditory SSDs such as the one used here offer several advantages over current visual-to-tactile SSDs. For example, while the Tongue Display Unit (TDU) visual-to-tactile SSD offers a potentially wide field-of-view, and has since being last tested for acuity increased its pixel grid from 144 to 324 pixels , which is likely to result in increased functional acuity, current models are far from the functional acuity demonstrated here, which is equivalent to ∼4500 pixels. Furthermore, beyond its relatively costly price, using the tongue to display visual information precludes its concurrent use for eating, drinking or talking, which will plausibly limit its use. Perhaps it may be more productive in the future to apply visual-to-tactile transformations to other skin surfaces, which may be less intrusive in every-day life. On the other hand visual-to-tactile SSDs offer better temporal resolution, improving detection of online motion and optic flow. Therefore, ultimately the optimal SSD will be one combining both auditory (e.g. through bone-conductance earphones, leaving the ears open) and tactile interfaces arriving from the same camera (see for instance a schematic diagram of such a proposed system in [40]).

These findings should thus also encourage the development of new SSDs with finer and additional visual detail, such as color (which is currently not provided in retinal prostheses) and direct depth cues. SSDs are also a unique research tool to study sensory and multisensory processing, developmental critical periods and adult plasticity, as well as cortical specialization in the blind visual cortex [31], especially for the processing of visual stimuli which require high-resolution ‘vision’, such as facial expressions and reading. Thus overall, our results suggest that auditory (and tactile) SSDs are both a valuable research tool and a potentially high resolution option in any clinical visual rehabilitation protocol.

## Supporting Information

Movie S1 **Utilization of high-resolution vision by a congenitally blind participant – identifying emotional facial expressions.** The video depicts a vOICe training session of one congenitally blind subject in which she is requested to identify emotional facial expressions of two individuals, on live, 3-dimentional faces. She is able to distinguish between a smiling, surprised and angry facial expression, and to identify the same emotional expressions on a novel face, exhibiting learning generalization. See also **Figure S1** depicting her possible approximate functional acuity.
(AVI)

Figure S1 **Deciphering facial expressions.** Illustration of the detail which can be conveyed by different current means of visual rehabilitation and that conveyed at the functional resolution perceived by our median participant, for the aim of detecting an emotional facial expression. Facial expression is perceivable using the vOICe SSD used here (see **Movie S1** depicting a congenitally blind participant conducting this task), but not in other current means of sight restoration.
(TIF)

## References

[1] WHO (2011). Fact Sheet N°.

[2] Dowling J (2008). Current and future prospects for optoelectronic retinal prostheses.. Eye.

[3] Weiland JD, Cho AK, Humayun MS (2011). Retinal prostheses: current clinical results and future needs.. Ophthalmology.

[4] Zrenner E, Bartz-Schmidt KU, Benav H, Besch D, Bruckmann A (2010). Subretinal electronic chips allow blind patients to read letters and combine them to words.. Proceedings of the Royal Society B: Biological Sciences.

[5] Bach-y-Rita P, Kercel SW (2003). Sensory substitution and the human-machine interface.. Trends Cogn Sci.

[6] Bach-y-Rita P (2004). Tactile sensory substitution studies.. Ann N Y Acad Sci.

[7] Chebat DR, Rainville C, Kupers R, Ptito M (2007). Tactile-‘visual’ acuity of the tongue in early blind individuals.. Neuroreport.

[8] Sampaio E, Maris S, Bach-y-Rita P (2001). Brain plasticity: ‘visual’ acuity of blind persons via the tongue.. Brain Res.

[9] Capelle C, Trullemans C, Arno P, Veraart C (1998). A real-time experimental prototype for enhancement of vision rehabilitation using auditory substitution.. IEEE Trans Biomed Eng.

[10] Renier L, De Volder AG (2010). Vision substitution and depth perception: Early blind subjects experience visual perspective through their ears.. Disability & Rehabilitation: Assistive Technology.

[11] Meijer PB (1992). An experimental system for auditory image representations.. IEEE Trans Biomed Eng.

[12] Lewis TL, Maurer D (2005). Multiple sensitive periods in human visual development: evidence from visually deprived children.. Dev Psychobiol.

[13] Wiesel TN, Hubel DH (1963). Single-Cell Responses in Striate Cortex of Kittens Deprived of Vision in One Eye.. J Neurophysiol.

[14] Wiesel TN, Hubel DH (1965). Comparison of the effects of unilateral and bilateral eye closure on cortical unit responses in kittens.. J Neurophysiol.

[15] Fine I, Wade AR, Brewer AA, May MG, Goodman DF (2003). Long-term deprivation affects visual perception and cortex.. Nat Neurosci.

[16] Gregory RL (2003). Seeing after blindness.. Nat Neurosci.

[17] Ostrovsky Y, Andalman A, Sinha P (2006). Vision following extended congenital blindness.. Psychol Sci.

[18] Ostrovsky Y, Meyers E, Ganesh S, Mathur U, Sinha P (2009). Visual Parsing After Recovery From Blindness.. Psychol Sci 20.

[19] Ackroyd C, Humphrey NK, Warrington EK (1974). Lasting effects of early blindness. A case study.. Q J Exp Psychol.

[20] Gregory RL, Wallace JG (1963). Recovery from early blindness: a case study.. Experimental Psychology Society, Monograph Supplement 2.

[21] Amedi A, Stern WM, Camprodon JA, Bermpohl F, Merabet L (2007). Shape conveyed by visual-to-auditory sensory substitution activates the lateral occipital complex.. Nat Neurosci.

[22] Auvray M, Hanneton S, O'Regan JK (2007). Learning to perceive with a visuo-auditory substitution system: Localisation and object recognition with ‘The vOICe’.. Perception.

[23] Proulx MJ, Stoerig P, Ludowig E, Knoll I (2008). Seeing ‘where’ through the ears: effects of learning-by-doing and long-term sensory deprivation on localization based on image-to-sound substitution.. PLoS ONE.

[24] Bailey IL, Lovie JE (1976). New design principles for visual acuity letter charts.. Am J Optom Physiol Opt.

[25] Stidwill D, Fletcher R (2010). Normal Binocular Vision: Theory, Investigation and Practical Aspects: John Wiley & Sons.

[26] Collignon O, Voss P, Lassonde M, Lepore F (2009). Cross-modal plasticity for the spatial processing of sounds in visually deprived subjects.. Exp Brain Res.

[27] Gougoux F, Lepore F, Lassonde M, Voss P, Zatorre RJ (2004). Neuropsychology: pitch discrimination in the early blind.. Nature.

[28] Hotting K, Roder B (2009). Auditory and auditory-tactile processing in congenitally blind humans.. Hear Res.

[29] Busskamp V, Duebel J, Balya D, Fradot M, Viney TJ (2010). Genetic reactivation of cone photoreceptors restores visual responses in retinitis pigmentosa.. Science.

[30] Locker M, Borday C, Perron M (2009). Stemness or not stemness? Current status and perspectives of adult retinal stem cells.. Curr Stem Cell Res Ther.

[31] Striem-Amit E, Dakwar O, Reich L, Amedi A (2011). The large-scale organization of “visual” streams emerges without visual experience Cereb Cortex.

[32] Reich L, Szwed M, Cohen L, Amedi A (2011). A ventral visual stream reading center independent of visual experience.. Curr Biol.

[33] Ptito M, Matteau I, Gjedde A, Kupers R (2009). Recruitment of the middle temporal area by tactile motion in congenital blindness.. Neuroreport.

[34] Matteau I, Kupers R, Ricciardi E, Pietrini P, Ptito M (2010). Beyond visual, aural and haptic movement perception: hMT+ is activated by electrotactile motion stimulation of the tongue in sighted and in congenitally blind individuals.. Brain Res Bull.

[35] Fiehler K, Burke M, Bien S, Roder B, Rosler F (2009). The human dorsal action control system develops in the absence of vision.. Cereb Cortex.

[36] Mahon BZ, Anzellotti S, Schwarzbach J, Zampini M, Caramazza A (2009). Category-Specific Organization in the Human Brain Does Not Require Visual Experience.. Neuron.

[37] Collignon O, Vandewalle G, Voss P, Albouy G, Charbonneau G (2011). Functional specialization for auditory-spatial processing in the occipital cortex of congenitally blind humans.. Proc Natl Acad Sci U S A.

[38] Renier LA, Anurova I, De Volder AG, Carlson S, VanMeter J (2010). Preserved functional specialization for spatial processing in the middle occipital gyrus of the early blind.. Neuron.

[39] Striem-Amit E, Bubic A, Amedi A, Murray MM, Wallace MT (2011). Neurophysiological mechanisms underlying plastic changes and rehabilitation following sensory loss in blindness and deafness.. Frontiers in the Neural Bases of Multisensory Processes.

[40] Reich L, Maidenbaum S (2012). The brain as a flexible task-machine: implications for visual rehabilitation using non-invasive vs. invasive approaches.. Current Opinion in Neurology.

