# Supplementary figures and images for: ‘Visual’ Acuity of the Congenitally Blind Using Visual-to-Auditory Sensory Substitution
Source: PLoS One. 2012 Mar 16;7(3):e33136. doi: 10.1371/journal.pone.0033136 (PMC3306374; doi:10.1371/journal.pone.0033136)

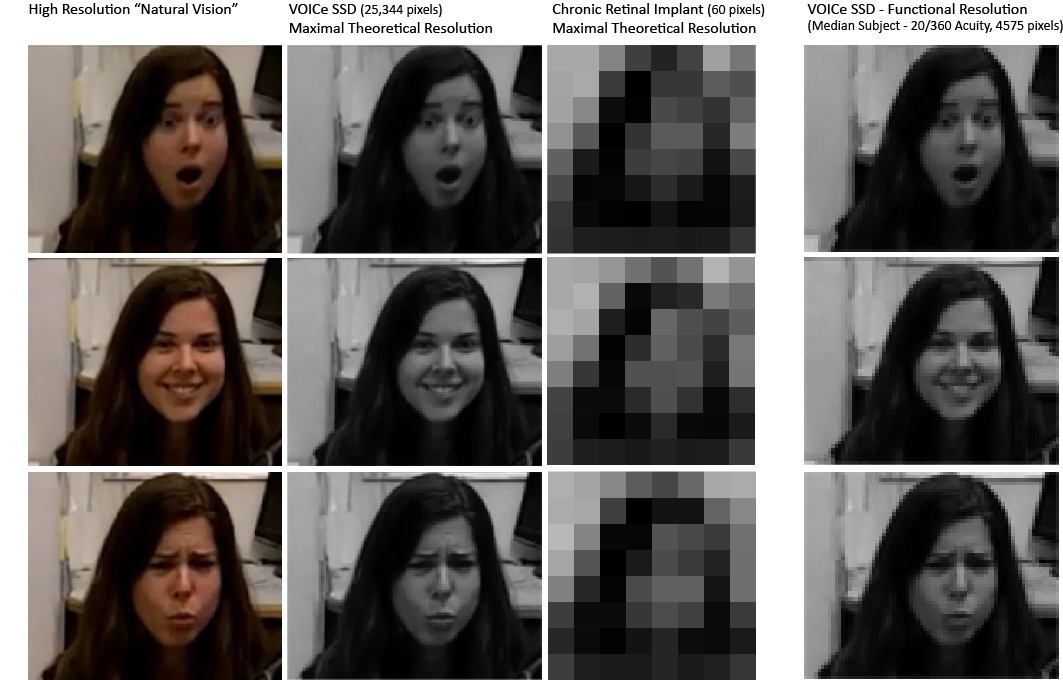

Supplement: Figure S1 — Deciphering facial expressions. Illustration of the detail which can be conveyed by different current means of visual rehabilitation and that conveyed at the functional resolution perceived by our median participant, for the aim of detecting an emotional facial expression. Facial expression is perceivable using the vOICe SSD used here (see Movie S1 depicting a congenitally blind participant conducting this task), but not in other current means of sight restoration. (TIF) [file pone.0033136.s002.tif]
